# Bubbler: A Novel Ultra-High Power Density Energy Harvesting Method Based on Reverse Electrowetting

**DOI:** 10.1038/srep16537

**Published:** 2015-11-16

**Authors:** Tsung-Hsing Hsu, Supone Manakasettharn, J. Ashley Taylor, Tom Krupenkin

**Affiliations:** 1 Department of Mechanical Engineering, University of Wisconsin-Madison, 1513 UniversityAvenue, Mechanical Engineering Building Room 2238, Madison, WI, 53706, USA

## Abstract

We have proposed and successfully demonstrated a novel approach to direct conversion of mechanical energy into electrical energy using microfluidics. The method combines previously demonstrated reverse electrowetting on dielectric (REWOD) phenomenon with the fast self-oscillating process of bubble growth and collapse. Fast bubble dynamics, used in conjunction with REWOD, provides a possibility to increase the generated power density by over an order of magnitude, as compared to the REWOD alone. This energy conversion approach is particularly well suited for energy harvesting applications and can enable effective coupling to a broad array of mechanical systems including such ubiquitous but difficult to utilize low-frequency energy sources as human and machine motion. The method can be scaled from a single micro cell with 10^−6^ W output to power cell arrays with a total power output in excess of 10 W. This makes the fabrication of small light-weight energy harvesting devices capable of producing a wide range of power outputs feasible.

Since late 1990 s portable electronic devices such as mobile phones, tablets, and laptops became an indispensable part of our daily life. However, powering mobile electronic devices is a challenge — electrical batteries often emerge as a critical bottleneck impeding portable electronics usage and development^1^. High-power mechanical energy harvesting can provide a valuable alternative to the use of batteries, but until now, its wide-spread adoption has been hampered by the lack of an appropriate mechanical-to-electrical energy conversion technology.

Recently, the authors have proposed a new method of mechanical-to-electrical energy conversion based on reverse electrowetting on dielectric (REWOD) phenomenon^2^. This method has a number of advantages over traditional conversion methods such as electromagnetic, piezoelectric, or electrostatic. Most notably, it has the potential to produce very high power densities, the ability to utilize a wide range of forces and displacements, and the ability to produce power at a broad range of currents and voltages (from several volts to tens of volts) without the need for up or down conversion.

As shown in Fig. 1(a), the conventional electrowetting on dielectric (EWOD) phenomenon can be viewed as a process of converting electrical energy into mechanical energy. When electrical voltage is applied between the conductive liquid droplet and the dielectric-coated electrode an electrostatic field builds up at the interface between the liquid and the electrode. In response the droplet moves to increase the area of contact between the droplet and the electrode in order to minimize the free energy of the system^3^. Thus, in the EWOD process electrical energy is partially converted into mechanical energy associated with the motion of a droplet. REWOD is in essence a reverse process to EOWD, as it converts mechanical energy into electrical energy. External mechanical force is used to periodically deform the droplet and thus induce the change in the area of contact between the droplet and the dielectric-coated electrode, which is connected to the bias voltage source, as shown in Fig. 1(a). This periodic change of the contact area induces the change in the electrical capacitance of the interface and forces electrical current to flow back and forth across the load resistor, producing energy.

Maximum achievable power density represents one of the most important characteristics of the energy conversion method in many applications. In energy harvesting high power density (ether by volume or by area) is very desirable, as it enables the fabrication of smaller and lighter devices that can be coupled to a broad range of energy sources. Power generated using REWOD can be maximized by two main methods. One is to increase the energy produced during each droplet oscillation, most notably by increasing the applied bias voltage, or by increasing the capacitance of the liquid-solid interface. However, this approach has an obvious limitation as a higher capacitance requires a thinner dielectric film which, in turn, restricts the maximum bias voltage that can be applied across the interface without the risk of electrical breakdown.

The second approach is to increase the oscillation frequency of the droplet. This not only increases the total power by producing more energy generating events per unit time, but also increases the energy produced for each oscillation due to the dynamics of the electrical charge transfer during the REWOD process^2^. This change in energy per cycle as a function of frequency for a specific bias voltage is illustrated in Fig. 1(b). Thus increasing the droplet oscillation frequency represents a particularly effective way to raise the power density generated by the REWOD. However, under regular circumstances, achieving a high droplet oscillation frequency requires an energy source capable of providing mechanical excitations with the comparably high frequency. Such sources of mechanical energy are not uncommon, with vibrations commonly generated by vehicles or operating machinery being a prime example. Unfortunately, a very broad range of potential mechanical energy sources ubiquitous in our environment is characterized by low to medium frequencies, from a fraction of a Hz to several Hz. Typical examples of these sources include human and machine motion, waves and tides, wind and temperature induced motion of building or other large structures, etc. All of those sources can generate high level of forces, and thus provide substantial mechanical power, but their characteristic frequency is too low to enable high energy harvesting power densities that the REWOD process is capable of achieving. One possible way to circumvent this issue is to utilize the energy harvesting approach that does not rely on the mechanical energy source to generate the required high frequency excitations but, instead, utilizes an internal fast oscillation process, which is independent from the mechanical energy source behavior. The challenge with this approach is to find a practical method of achieving such fast internal oscillation dynamics without resorting to complex mechanical or hydraulic systems.

The “bubbler” method provides an elegant solution to this problem by combining the REWOD phenomenon with a high frequency oscillation process, naturally occurring during the bubble growth and collapse. This makes it well suited for extracting energy from a variety of mechanical energy sources characterized by a wide range of frequencies and allows one to dramatically increase the generated power, which is proportional to the product of the oscillation frequency and the energy produced during each oscillation.

## Results

### Bubbler concept

The bubbler conceptual design is very simple and is shown in Fig. 2. The core of the bubbler contains no moving mechanical parts and consists of only three major elements: (i) a REWOD chip with an array of dielectric-coated circular electrodes, each electrode having a hole in the center, (ii) a thin membrane separated from the REWOD chip by a small gap, and (iii) a top plate, which serves to support the membrane and allows the dielectric fluid to escape. The gap between the REWOD chip and the membrane is filled with the conductive liquid, which does not wet the membrane and thus cannot penetrate it, as shown in Fig. 2(b). A pressurized dielectric fluid (e.g. air, inert gas, or a dielectric liquid) is supplied through the holes in the chip, causing dielectric bubbles to grow on top of each circular electrode. The growing bubbles displace the conductive liquid and thus reduce the area of overlap between the conductive liquid and the electrodes, inducing electrical current in the circuit. Each bubble continues to grow until it becomes large enough to touch the membrane. At this point the dielectric fluid starts to escape through the membrane, causing a rapid bubble collapse. The frequency, with which the bubble growth and collapse process repeats itself, is controlled by the size of the gap between the membrane and the electrode, by the viscosities of the fluids, and by the pressures applied to the dielectric fluid and the conductive liquid. A video with the animation of the bubbler operation is provided in the Supplementary Materials (see Supplementary Video 1). A detailed description of the bubbler device fabrication is given in the Methods section.

The bubbler simple design allows it to be implemented in a wide variety of shapes and sizes, and the bubbler output power can be easily scaled by appropriately adjusting the size of the bubble array. The bubble self-oscillation occurs naturally as long as there is an external pressure applied to the energy harvesting device thus allowing effective coupling of the device to mechanical energy sources characterized by a very wide range of forces, displacements, and frequencies.

This is particularly true for low-frequency energy sources, such as human, machine, or buildings motion. Very low frequencies (from several Hz to less than 1 Hz) typical for such motion can severely hamper energy harvesting by dramatically reducing the number of energy generating events per unit time. However, since the bubbler energy generating events frequency is determined by the frequency of the bubble self-oscillation, which is independent of the frequency associated with the energy source, the energy density of the bubbler method remains high.

For example, a simple bubbler device, which can be integrated into footwear to enable energy harvesting from human locomotion (about 1 Hz frequency) is shown in Fig. 3. In this case the bubbler chip is located between two chambers filled with the pressurized gas. During the heel strike the top elastic chamber is compressed and the gas is displaced through the REWOD chip inducing many thousands of bubble oscillations, each of which converts a portion of mechanical energy of the heel strike into electrical energy. During the toe-off process the compressed gas flows from the bottom chamber back to the top chamber through an auxiliary bypass check valve completing the cycle. Estimations, as discussed below, show that such a device can generate about 1 W of usable electrical power. A similar device, which harvests energy from machine motion and is capable of generating over 5 W of power is discussed in Supplementary Material.

The oscillatory flow induced by the bubble growth and collapse process unavoidably causes mechanical energy dissipation due to viscous forces. This, in turn, affects the bubbler energy conversion efficiency. The rate of energy dissipation depends strongly on the details of the bubbler geometry. The major contributors are the gap thickness, the bubble size, and the inlet-hole diameter, as well as the fluid viscosity and the bubble oscillation frequency. Estimations, as discussed in the Results section, show that the energy conversion efficiency of the bubbler setup used in the current experiment is on the order of several percent. This, however, does not represent the intrinsic limitations of the technology, as the current experimental setup has not been optimized for efficiency. We also would like to note that in many situations the energy conversion efficiency is not the most important characteristic of the energy harvesting technology. Because for a wide range of applications the power absorbed by the harvester is much smaller than the power of the energy source itself, the behavior of the source is not influenced by the harvester in any appreciable way. Under such circumstances the most important characteristic of the harvesting technology is its power density as it directly determines the maximum power that can be generated by the harvester of a particular size. In view of this we have concentrated in this work on the investigation of the various parameters that affect the power density of the bubbler method, leaving investigation of the bubbler conversion efficiency for a separate work.

### Theoretical model and experimental results

The bubbler approach is predicated on the ability to achieve a stable and predictable bubble self-oscillation process. Thus we paid special attention to theoretical and experimental investigation of the bubble growth and collapse dynamics. To this end we combined experimental studies of the bubble behavior with the computational fluid dynamics (CFD) modeling of the bubble growth and collapse process. The laminar two phase flow CFD model was used to obtain the numerical solution for the fluid velocity distribution and the bubble interface dynamics. The dielectric fluid and the conductive liquid used in most of the simulations were air and mercury respectively. Our previous experiments^2^ demonstrated that the REWOD process works best when room-temperature liquid metals are used as a conductive liquid. In particular, mercury and a gallium-indium alloy called Galinstan were shown to be well suited for use with REWOD^2^. In this work we utilized mercury due to its chemical stability and resistance to oxidation. In potential commercial applications mercury is undesirable due to its toxicity and the use of gallium-indium alloys might be preferred. The applied gauge pressures at the air inlet (i.e. the hole through the electrode) and at the mercury-filled gap between the electrode and the membrane were kept constant during the oscillation process. The simulation domain (electrode diameter) was varied between 1,000 μm and 1,200 μm. The diameter of the air inlet was varied between 140 μm and 200 μm. The gap between the electrode and the membrane was varied between 100 μm and 130 μm. The details of the CFD modeling procedure are available in the Supplementary Materials. The four major steps of the bubble oscillation process, which were observed in the model, are shown in Fig. 4(a–d) (also see Supplementary Video 2). At the beginning of the oscillation cycle the compressed air supplied through the inlet initiates the bubble growth (see Fig. 4(a)), which continues until the bubble touches the membrane, breaking the remaining mercury film and causing it to rapidly retract towards the edge of the electrode, as shown in Fig. 4(b,c). At this point the bubble takes the pancake shape and starts collapsing towards the center of the electrode, rapidly completing the self-oscillation cycle, as shown in Fig. 4(d).

The typical results obtained by CFD modeling for the time dependence of the footprint area of the bubble are shown in Fig. 4(e). The dependence of the bubble oscillation period and the maximum bubble expansion diameter on the size of the gap between the electrode and the membrane are shown in Fig. 5(c) and Fig. 5(b) respectively. The gap size exerts a profound influence on the bubble dynamics. As one can see from Fig. 5(b,c) the predicted bubble oscillation period appears to be a liner function of the gap size, while the predicted maximum bubble expansion diameter appear to be a quadratic function of the gap size.

The dependence of the bubble oscillation period and the maximum bubble expansion diameter on the inlet cross-sectional area is shown in Fig. 5(a). As one can see the predicted influence of the inlet cross-sectional area on the bubble oscillation dynamics is quite limited, as compared to the influence of the gap size. The predicted bubble oscillation period appears to be inversely proportional to the square root of the inlet cross-sectional area, i.e. inversely proportional to the inlet diameter. The predicted maximum bubble expansion diameter does not appear to show any obvious dependence on the inlet cross-sectional area.

The dependence of the bubble oscillation period on the mercury and air inlet pressures is shown in Fig. 5(d). The mercury pressure controls the rate of the bubble collapse process, while the difference between the air pressure and the mercury pressure controls the rate of the bubble growth process. By increasing the air inlet pressure one can increase the bubble oscillation frequency, driving the oscillation period below 0.5 ms, which corresponds to the frequencies in excess of 2 kHz, as shown in Fig. 5(d).

The predictions of the CFD model were supported by the experimental measurements of the intensity of the light penetrating through the bubbler assembly. This simple method, the details of which are illustrated in Fig. 6, allows experimental observation of the bubbler oscillation dynamics at high frequencies (see Supplementary Video 3). The observed light intensity is expected to scale linearly with the area of contact between the bubble and the membrane, as shown in Fig. 6(a–c). The typical result for the air bubble self-oscillations are shown in Fig. 4(f). As one can see the variation of the relative light intensity with time is in good qualitative agreement with the theoretical curve for the time dependence of the footprint area of the bubble, as shown in Fig. 4(e). The experimentally obtained peaks of the light intensity have noticeable variability, which, in our opinion, can be attributed mainly to the random deviation of the bubble footprint shape from the ideal circle, caused by the occasional pining of the mercury contact line by the imperfections of the electrode surface. These deviations produce random variations in the bubble footprint area, which, in turn, result in the variation of the registered light intensity. In addition to that the model setup invariably includes some simplifying assumptions. In particular the calculated results for the bubble footprint area shown in Fig. 4(e) are based on the assumption that the air pressure inside the bubbler chip is equal to the air pressure in the air supply line, which might not be exactly true. This might cause some additional difference between the model and the experimental results.

The experimentally observed dependence of the maximum bubble expansion diameter on the gap thickness is in good agreement with the theoretical predictions as shown in Fig 5(b). The experimentally observed dependence of the bubble oscillation period on the gap size appear to be stronger than the theoretical prediction, with the oscillation period proportional to the fourth power the gap size, as shown in Fig. 5(c). We believe that this deviation might be related to the limitations on the maximum air flow rate that the current experimental setup can support. A detailed investigation of this topic is currently underway. Similarly, the experimentally observed dependence of the bubble oscillation period on the inlet cross-sectional area has the same qualitative behavior as the theoretical prediction, but differs from it numerically, see Fig. 5(a). Again, we believe that this deviation might be caused by the air supply limitations mentioned above.

Dielectric fluids with a wide range of viscosities such as air, water, and hexane, have been tested in this work. The increase in the viscosity of the dielectric fluid leads to dramatic decrease in the bubble oscillation frequency. Thus the replacement of air (dynamic viscosity of 0.019 cP) by water (dynamic viscosity of 0.9 cP) results in the change of the oscillation frequency by over an order of magnitude, from 300 Hz for air to 17 Hz for water. A more detailed experimental investigation of the dependence of the bubble dynamics on the geometry of the system and the materials involved is currently underway and the results will be published elsewhere.

**Figure 1:**
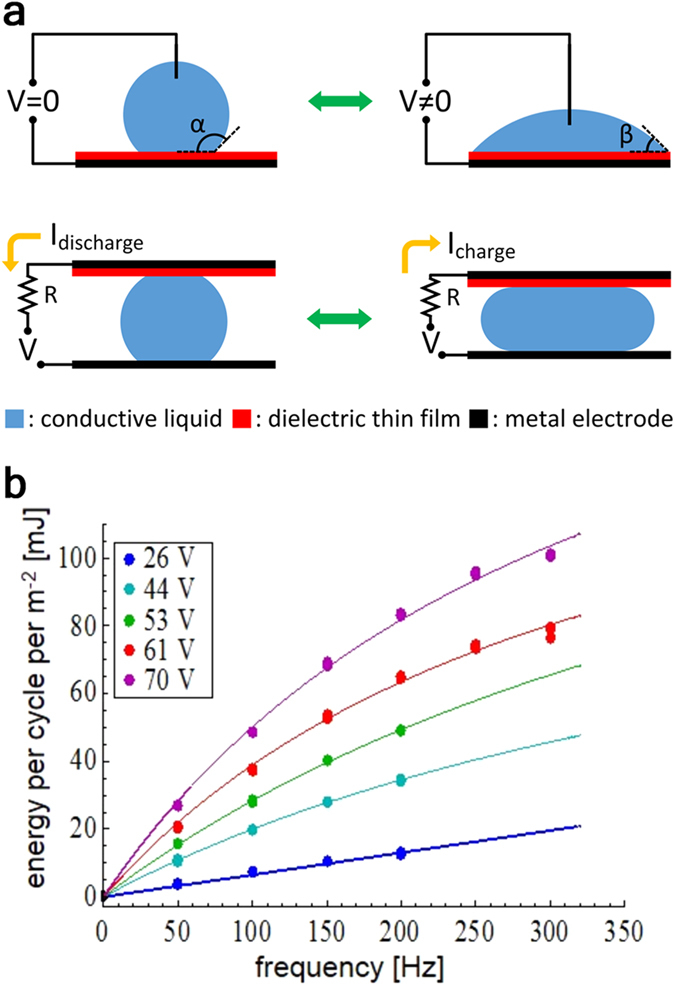
EWOD and REWOD concepts. (**a**) Schematics of the EWOD and REWOD process. The energy is generated during the contact area change. (**b**) Typical results for the energy generated per unit area per one oscillation cycle as a function of the oscillation frequency. Dots represent the actual experimental data; solid lines represent the best fit^2^.

**Figure 2:**
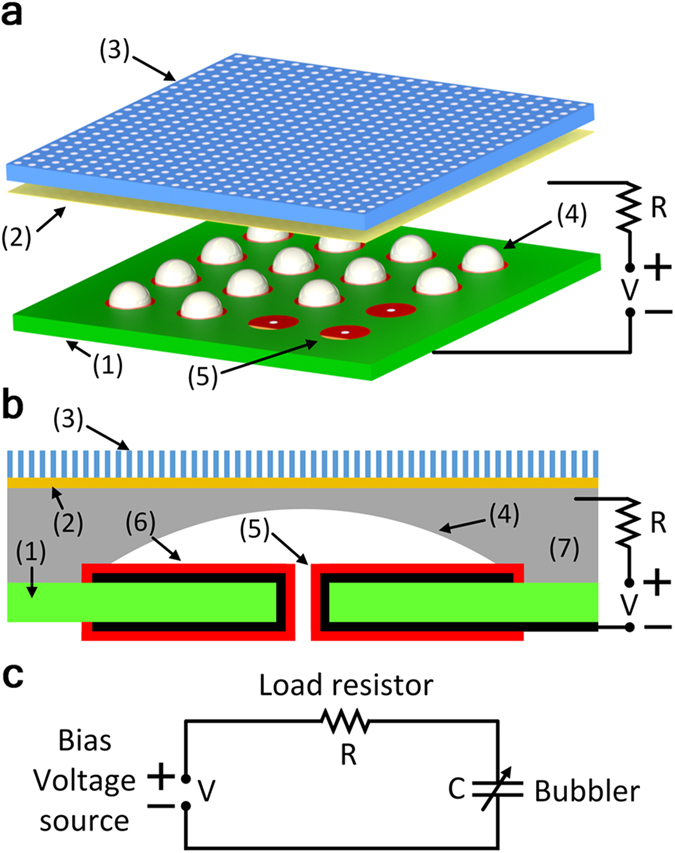
The bubbler concept. (**a**) Bubbler conceptual design: (1) indicates REWOD chip, (2) membrane, (3) top plate, (4) an array of bubbles, and (5) an array of electrodes. (**b**) Schematics of a simplified single-electrode device used in the experiment: (1) represents REWOD chip, (2) membrane, (3) top plate, (4) bubble, (5) metal electrode, (6) dielectric coating, and (7) conductive liquid. (**c**) Equivalent electrical circuit for a single-electrode device.

The power output of the bubbler can be predicted by analyzing the electrical circuit shown in Fig. 2(c). The circuit behavior is described by the following equation:


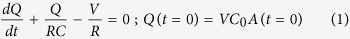


where *Q* is the charge on the electrode, *R* is the load impedance, *C* is the time-dependent capacitance of the mercury-electrode interface, *C*_0_ is the capacitance of the mercury-electrode interface per unit area, *V* is the bias voltage, and *t* is time. The capacitance of the mercury-electrode interface is *AC*_0_, where *A* is the area of contact between the mercury and the electrode, see ref. 2 for a more detailed discussion. In the current work the value of *C*_0_ was about 0.84 nF mm^−2^. The area *A* oscillates between zero and the electrode area as the result of the periodic bubble growth and collapse process. The dependence of area *A* on time *t* was obtained from the CFD model as discussed above. The resulting function *A*(*t*) was substituted in equation (1) and the solutions for the time-dependent charge *Q*(*t*) and the electrical current *I*(*t*) = d*Q*/d*t* were obtained by numerically solving equation (1). The typical results for the current *I*(*t*) and the generated power *P*(*t*) = *RI*^2^ for the bias voltage of 4.5 V are shown in Fig. 7(a,b). The energy produced by the bubble during one oscillation was obtained by integrating *P*(*t*) over one oscillation period. The obtained results for the maximum peak power density at 4.5 V bias voltage were about 80 Wm^−2^ and the energy density per one oscillation was 16 mJm^−2^, which is consistent with the previously demonstrated REWOD results shown in Fig. 1(c).

In order to experimentally investigate the REWOD-based energy generation process the electrode and the mercury were connected to a constant bias voltage source (i.e. electrochemical battery) and the voltage induced across the load resistor during bubble self-oscillation was measured as a function of time. The electrodes were produced out of tantalum, which was selected because of superior properties of its anodic oxide, most notably a high dielectric constant equal to 25 and a high breakdown electric field^4^,^5^,^6^,^7^. The tantalum film was deposited on both sides of the quartz REWOD chip in order to bridge the laser drilled air inlet hole through the chip and provide an electrical connection between the back side of the chip and a circular REWOD electrode on the front side of the chip. Tantalum electrode was then anodized to obtain a 200 nm thick tantalum pentoxide film covering the electrode. On top of the tantalum pentoxide a 6 nm layer of Cytop fluoropolymer was deposited using spin coating. A detailed description of the bubbler device fabrication is given in the Methods section below.

The typical results for the current flowing across the load resistor during the bubbler operation are shown in Fig. 7(c–f) for 4.5 V and 3.0 V bias voltages. As expected, the bubble growth and collapse induces spikes of current. It is quite clear that there is a good quantitative agreement between the experimentally obtained dependence of electrical current on time, which is shown in Fig. 7(c) and the theoretically predicted curves, which are shown in Fig 7(a). The observed random variation in the peak current values can be attributed to the same factor as variations in the light intensity peaks discussed above, i.e. random bobble footprint asymmetry.

The experimental areal power density plot is shown in Fig. 7(d), and it is in good agreement with the theoretical prediction shown in Fig. 7(b). The experimentally achieved peak power density at 4.5 V bias voltage is quite high and approaches 100 Wm^−2^, which is similar to the power densities obtained by the authors in the previous REWOD experiments^2^. The energy produced by the bubble during one oscillation was obtained by integrating *P*(*t*) over one oscillation period and is equal to about 13 nJ for the curve shown in Fig. 7(c). By comparing this value with the viscous energy dissipation per cycle estimated from the modeling data (about 400 nJ) one can estimate the energy conversion efficiency of the current setup to be about 3.5%.

## Discussion

The developed CFD model of the bubble self-oscillation process taken in conjunction with equation (1) can be used to predict the performance of the bubbler method under various conditions. As was already discussed above the bubbler power density can be further increased by increasing the bias voltage or further increasing the oscillation frequency. The predicted power densities for the higher frequencies and higher bias voltages than those used in the experiment are shown in Fig. 8. The data show that at bias voltages above 50 V and oscillation frequencies above 2 kHz the projected average power density can exceed 10 kWm^−2^, which is an order of magnitude more than the maximum power density expected from the REWOD method alone^2^, i.e. without it being combined with the bubble self-oscillation process. The maximum power that can be experimentally obtained using the bubbler approach is likely to be below the theoretical predictions, with the dielectric film quality and its electrical breakdown stress likely to be the main limiting factors. This topic is the subject of the ongoing research and the results will be published elsewhere.

**Figure 3:**
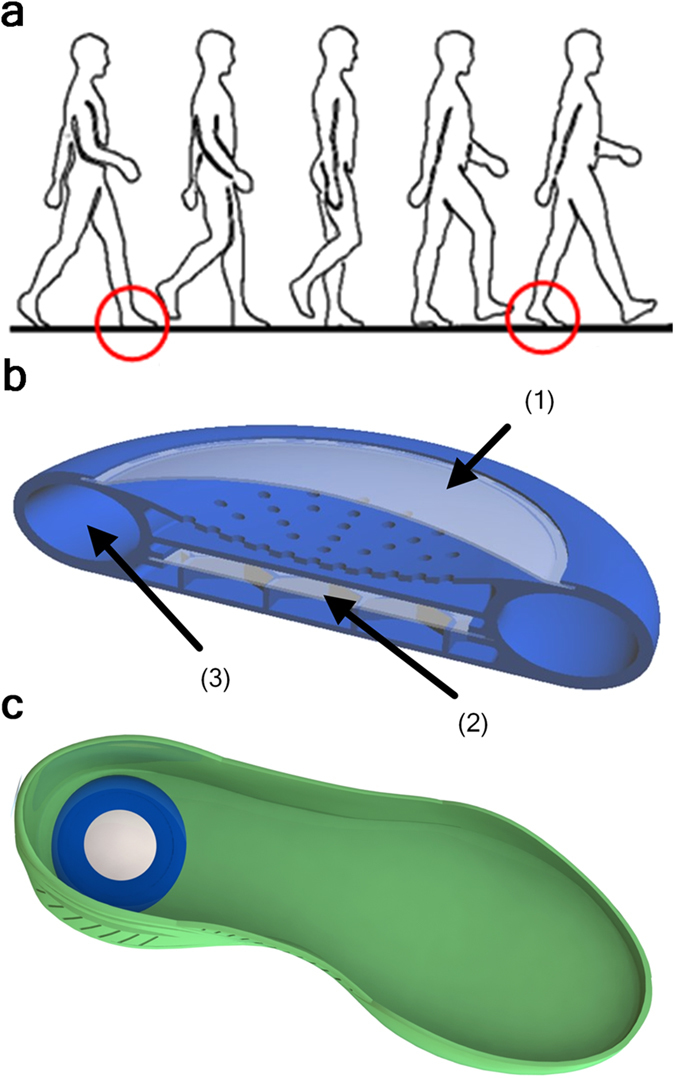
Energy harvesting from human locomotion. (**a**) The schematic of human locomotion: red circles indicate the heel-strike and toe-off. (**b**) Cross-section of footwear-integrated energy harvester based on the bubbler approach. Bypass check valve is not shown. (1) represents top flexible chamber filled with compressed inert gas, (2) REWOD chip, and (3) Bottom gas chamber. (**c**) Schematic representation of the harvester device embedded in the footwear sole.

**Figure 4:**
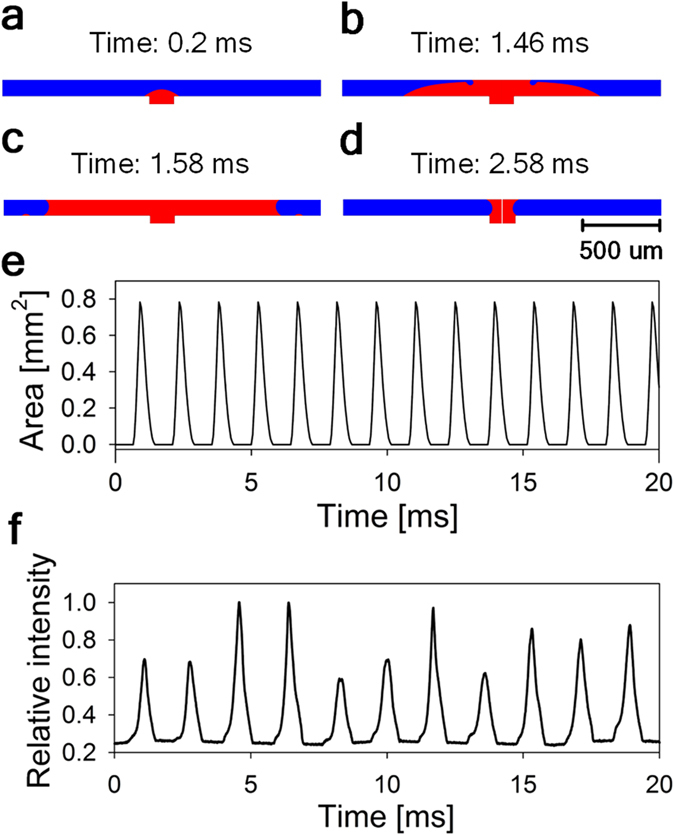
Theoretical and experimental investigation of the bubble dynamics. (**a–d**) CFD simulation of the bubble growth and collapse process. Red color indicates the volume occupied by the air, and blue color represents the volume occupied by the mercury. (**e**) The results of the CFD modeling for the area occupied by air. For this simulation the gauge pressures at the air inlet (P_Air_ - P_Hg_) and at the mercury (P_Hg_) were 0.08 bar and 0.08 bar, respectively. The predicted oscillation period was about 1.4 ms. (**f**) Experimental results for the relative light intensity vs. time. The obtained oscillation period is about 1.7 ms.

**Figure 5:**
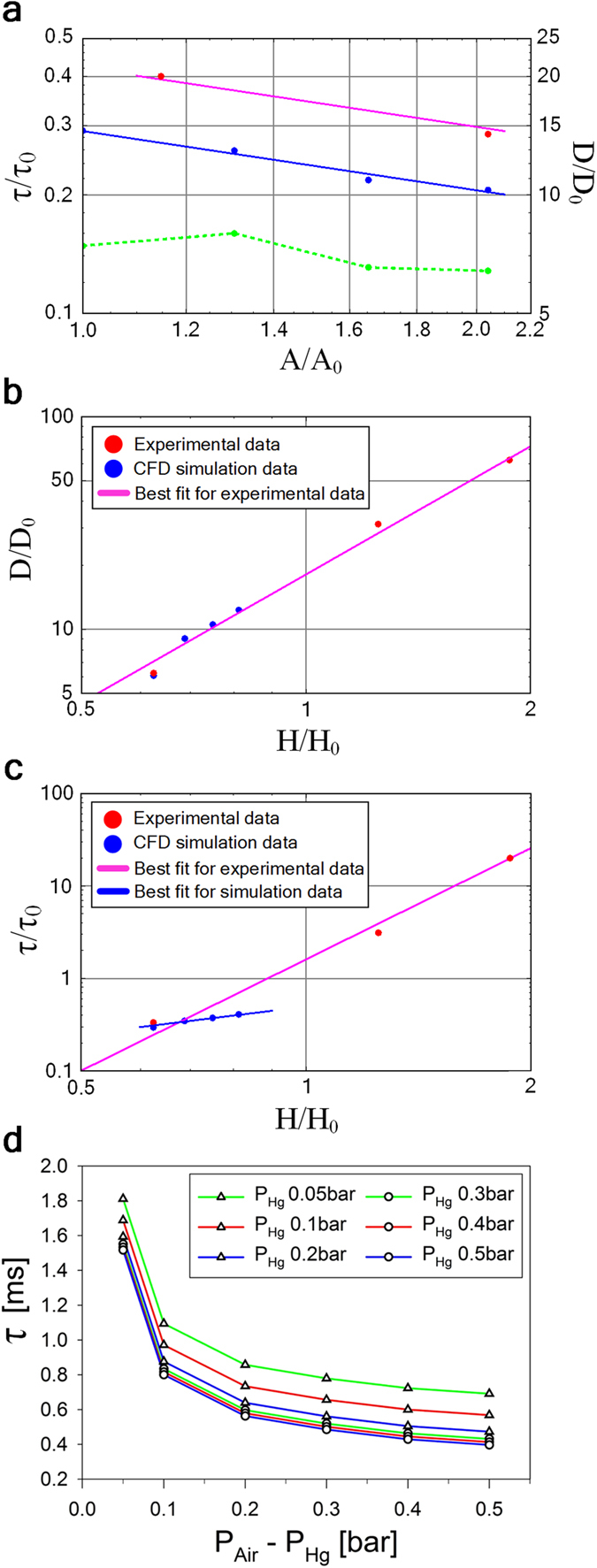
Theoretical investigation of the bubble growth and collapse process. (**a**) CFD simulation and experimental results for the bubble oscillation period τ and the maximum bubble expansion diameter D as a function of the inlet cross-sectional area A. Blue dots represent simulation results for the bubble oscillation period τ , while the blue line represents the best fit of the form τ ~ A^−1/2^. Red dots represent experimental results for the bubble oscillation period τ, while the magenta line represents the best fit of the form τ ~ A^−1/2^. Green dots represent simulation results for the maximum bubble expansion diameter D. The dashed green line is to guide the eye only. The following values are used: τ_0_ = 10 ms, D_0_ = 0.16 mm, A_0_ = 0.0154 mm^2^. (**b**) CFD simulation and experimental results for the maximum bubble expansion diameter D as a function of the gap *H* between the membrane and the electrode. Blue dots represent simulation results for the maximum bubble expansion diameter D. Red dots represent experimental results for the maximum bubble expansion diameter D, while the magenta line represents the best fit of the form D ~ *H*^2^. The following values are used: τ_0_ = 10 ms, H_0_ = 0.16 mm. (**c**) CFD simulation and experimental results for the bubble oscillation period τ as a function of the gap *H* between the membrane and the electrode. Blue dots represent simulation results for the bubble oscillation period τ , while the blue line represents the best fit of the form τ ~ *H*. Red dots represent experimental results for the bubble oscillation period τ , while the magenta line represents the best fit of the form τ ~ *H*^4^. The following values are used: H_0_ = D_0_ = 0.16 mm. (**d**) The dependence of the bubble oscillation period τ on the mercury and air inlet pressures, P_Hg_ and P_Air_ respectively. The solid lines are to guide the eye only.

**Figure 6:**
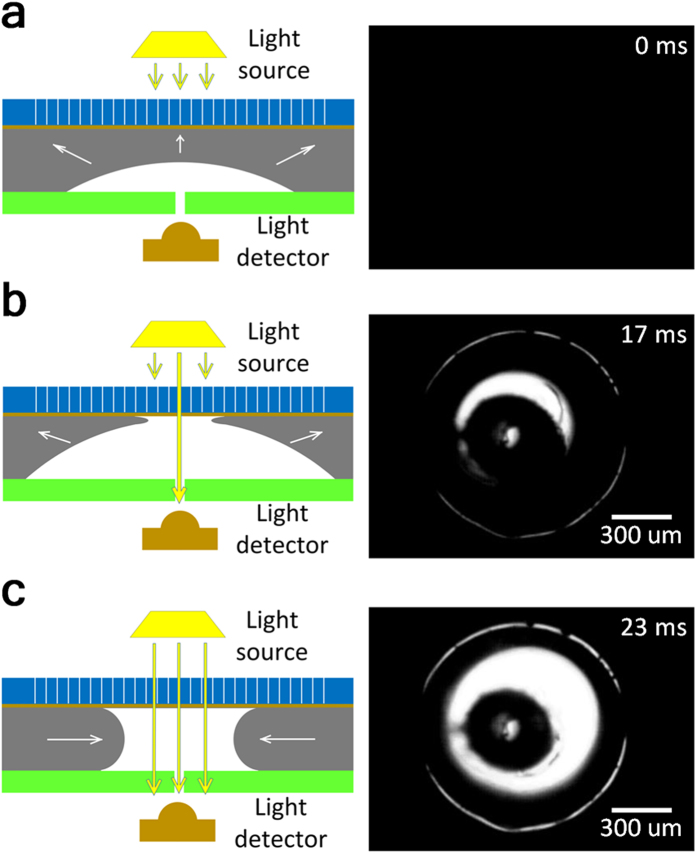
Experimental investigation of the bubble growth and collapse process. The schematics of the experiment is shown on the left, and the actual image of the light source as seen by the high-speed camera positioned at the light detector spot is shown on the right. The black central spot in the actual image indicates the tube connecting to the pressurized dielectric fluid. Water was used as a dielectric fluid in this experiment. (**a**) indicates the initial stage of bubble growth, (**b**) final stage of bubble growth, and (**c**) bubble collapse.

**Figure 7:**
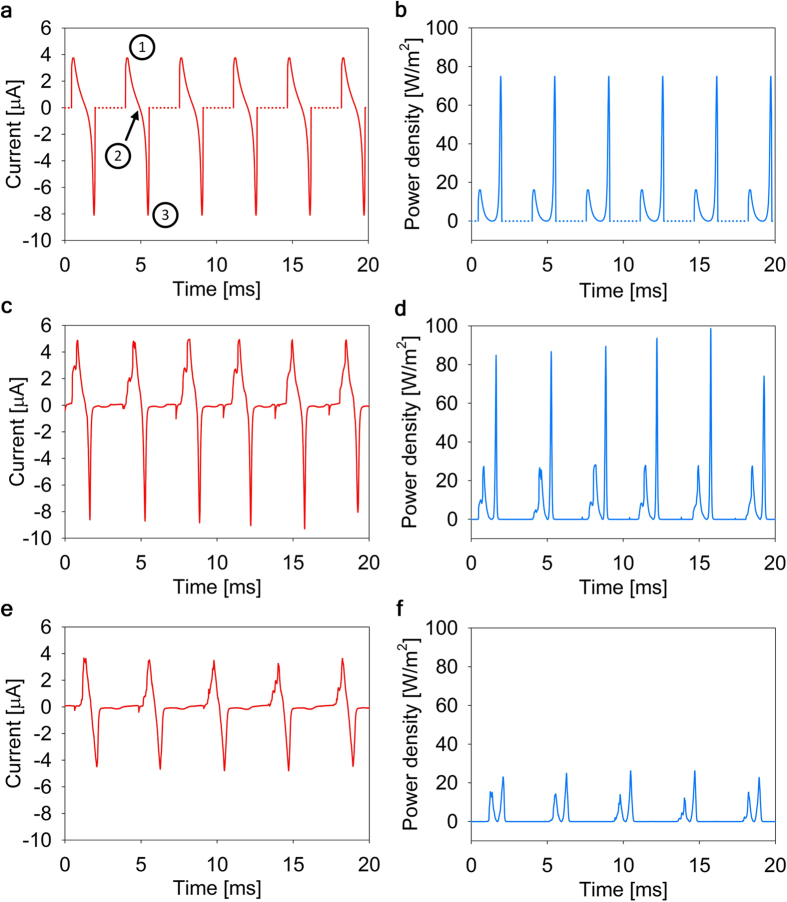
Theoretical and experimental investigation of the bubbler electrical performance. (**a**) Theoretical prediction of the current flowing through the load resistor. (1) denotes the bubble collapse process, (2) the beginning of the bubble growth process, and (3) the final stage of the growth process where the bubble footprint exceeds the electrode. (**b**) Theoretical prediction of the power generated on the load resistor. (**c**) Experimental results for the current flowing through the load resistor at bias voltage of 4.5 V. (**d**) Experimental results for the power generated on the load resistor at bias voltage of 4.5 V. (**e**) Experimental results for the current flowing through the load resistor at bias voltage of 3 V. (**f**) Experimental results for the power generated on the load resistor at bias voltage of 3 V.

**Figure 8:**
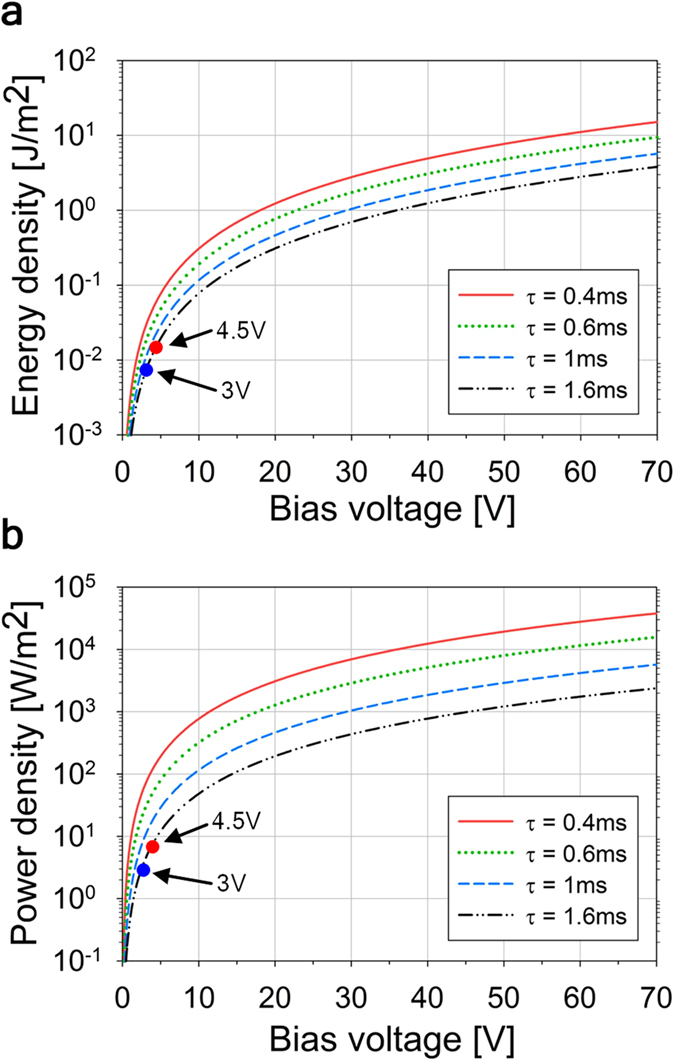
Bubbler power generation predictions. (**a**) Projected energy per unit area obtained during one oscillation event as a function of the oscillation period τ and the applied bias voltage. (**b**) Projected average power per unit area as a function of the oscillation period τ and the applied bias voltage. The large red and blue dots represent the obtained experimental results.

The ability to achieve high power densities makes compact energy harvesting devices with the power output on the level of several watts quite feasible. In particular, the footwear embedded harvester device discussed above and shown in Fig. 3 is designed to operate at 30 V bias voltage and the bubble self-oscillation frequency of 2 kHz. The device incorporates a 40 mm × 40 mm size REWOD chip which carries an array of 800 electrodes with 1 mm diameter and with the total area of 628 mm^2^. The results shown in Fig. 8(b) can be used to estimate the expected average power output of the harvester as about 1 Watt, based on 0.25 s heel strike duration and one step per second walking cadence.

In summary, we have proposed and experimentally demonstrated the “bubbler” – a novel approach to direct conversion of mechanical energy into electrical energy. The main advantage of the bubbler approach is its ability to combine a high frequency self-oscillation process, naturally occurring during the air bubble growth and collapse, with the REWOD-based energy conversion process, which is capable of producing large quantities of energy per each oscillation. This allows one to maximize the produced power, which is proportional to the product of the oscillation frequency and the energy produced during each oscillation. Experimentally obtained power density was about 100 Wm^−2^, and theoretical estimates show that power densities in excess of 10 kWm^−2^ can be expected under the right conditions. The bubble self-oscillation occurs naturally as long as there is an external pressure applied to the device thus allowing effective coupling of the device to mechanical energy sources possessing a wide range of forces, displacements, and frequencies. The simplicity and flexibility of the bubbler approach makes it particularly suitable for the energy harvesting applications, especially for cases where direct coupling to low-frequency, high-force mechanical energy sources, such as human, machine, or buildings motion is desired. We hope that this novel approach will lead to the development of a broad range of REWOD-based energy harvesting devices.

## Methods

### Fabrication of REWOD chip

**Figure 9:**
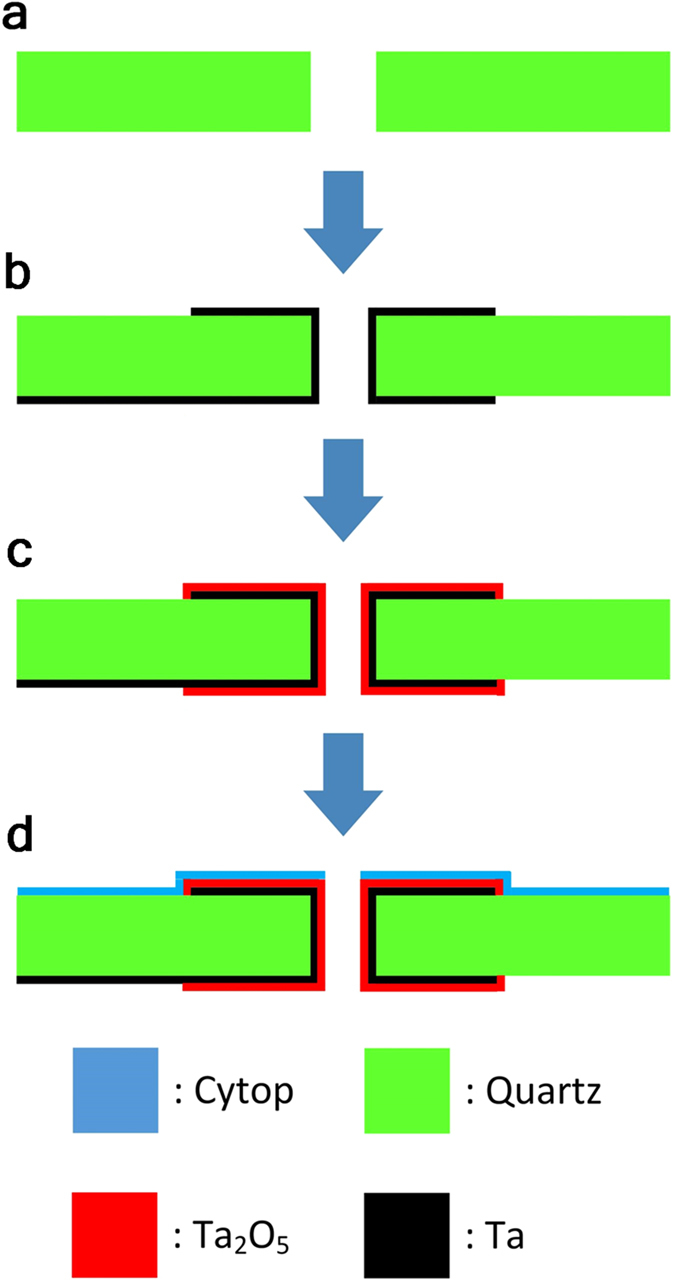
The REWOD chip fabrication process. (**a**) shows the 10 mm × 10 mm quartz substrate with a laser drilled hole in the center, (**b**) sputter deposition of tantalum on front and back side which electrically bridges the center hole, (**c**) anodization of tantalum film in citric acid, and (**d**) cytop spin-coated on tantalum pentoxide.

The REWOD chips were fabricated using a 300 μm thick 2 inch diameter quartz wafer acquired from University Wafer, USA. The wafer was diced into 10 mm by 10 mm square chips. At the center of each sample a 160 μm diameter hole was fabricated by using laser drilling (performed by K Jet Laser Technologies, INC, Taiwan). A 1 μm thick Ta layer was sputter deposited on both sides of the chip using a shadow mask to define the pattern. The Ta layer was deposited using a CVC 601 Sputter System with a power of 1.0 kW. The Ta layer was also deposited on the walls of the hole to form an electrical connection between the front and the backside of the chip. To increase the breakdown voltage of tantalum pentoxide formed after anodization, a mixture of argon and nitrogen (Ar/N_2_ ratio 20/1.45 sccm) was used for the sputter deposition^6^. The Ta layer was then anodized using constant electrical current (2.6 × 10^−4^ A/cm^2^) in 0.01 wt% citric acid aqueous solution to form a thin Ta_2_O_5_ layer of 200 nm on top of the Ta. The Ta_2_O_5_ thickness was determined by the anodization voltage^6^ yielding about 2 nm/V. Finally, a Cytop fluoropolymer, acquired from Asahi Glass Company, Japan, was used to deposit a thin fluoropolymer film on the Ta_2_O_5_. A Cytop solution, consisting of 0.5 wt % concentration in perflurohexane^8^, was spin-coated on top of the Ta_2_O_5_ layer and cured at 180 °C for 1 h. A summary of the major steps in the REWOD chip fabrication is shown schematically in Fig. 9.

### Experimental Setup

**Figure 10:**
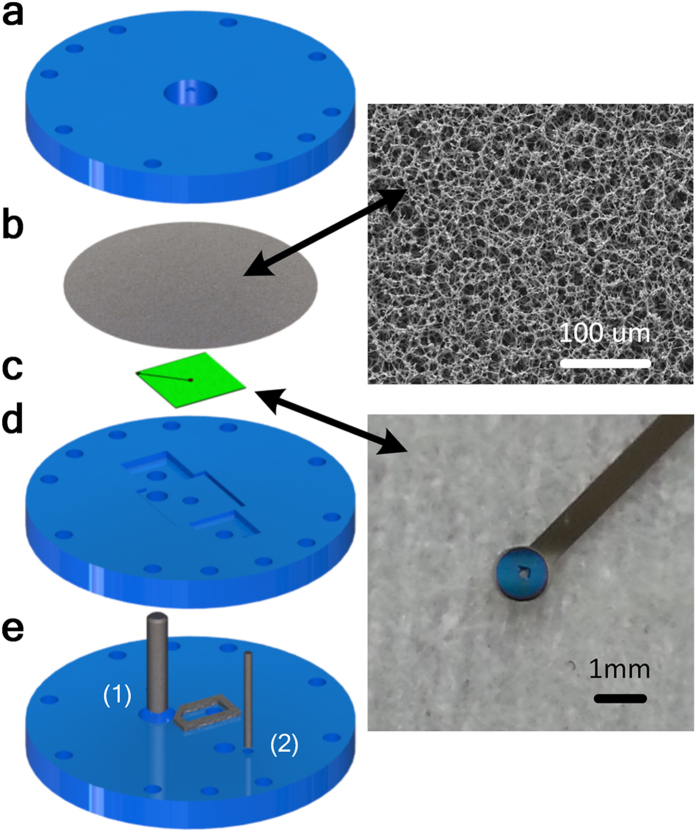
Bubbler experimental setup. (**a**) shows the top housing plate with an array of tiny holes, (**b**) membrane, (**c**) REWOD chip, (**d**) mid housing plate which serves as the support for REWOD chip and the reservoir for the conductive liquid, (**e**) bottom housing plate with the connector for the pressurized dielectric fluid. (1) denotes electrical connector to REWOD chip and (2) the electrical connector to conductive liquid.

Figure 10 shows a blow-up view of the bubbler experimental setup. The bubbler is composed of three housing plates, a membrane, a REWOD chip and several electrical connectors. A CNC milling machine was used to make the housing plates, and the mixed cellulose ester (MCE) membrane with the pore size of about 8 μm was obtained from Sterlitech Corporation, USA. The thickness of the gap between the membrane and the REWOD chip varied from about 100 μm to 200 μm, and the inlet pressure required to initiate the bubble growth process was about 0.05 bar. A 0.877 MΩ resistor was used as a load. Stainless steel rods were selected as the electrical connection material because of their chemical stability in mercury, which was used as the conductive liquid in this experimental setup.

## Additional Information

**How to cite this article**: Hsu, T.-H. *et al.* Bubbler: A Novel Ultra-High Power Density Energy Harvesting Method Based on Reverse Electrowetting. *Sci. Rep.* **5**, 16537; doi: 10.1038/srep16537 (2015).

## Supplementary Material

Supplementary Information

Supplementary Video 1

Supplementary Video 2

Supplementary Video 3
