# Supplementary material for: Bubbler: A Novel Ultra-High Power Density Energy Harvesting Method Based on Reverse Electrowetting
Source: Sci Rep. 2015 Nov 16;5:16537. doi: 10.1038/srep16537 (PMC4645099; doi:10.1038/srep16537)
Supplement: Supplementary Information [file srep16537-s1.docx]

Bubbler: A Novel Ultra-High Power Density Energy Harvesting Method Based on Reverse Electrowetting

Tsung-Hsing Hsu, Supone Manakasettharn, J. Ashley Taylor, and *Tom Krupenkin

*Department of Mechanical Engineering, University of Wisconsin-Madison*

*1513 University Avenue, Mechanical Engineering Building Room 2238, Madison, WI, 53706, USA*

**Tel: 608 890 1948 and e-mail:* [*tnk@engr.wisc.edu*](mailto:tnk@engr.wisc.edu)

Supplementary Material

**CFD simulations**

CFD modeling was performed using COMSOL Multiphysics package^1^. A two-phase laminar flow model was used to model both bubble growth and collapse processes. A 2D axisymmetric mode was applied in order to make use of the axial symmetry of the bubble geometry. Typical meshes used to model the bubble growth and collapse processes are shown in Fig. 1 and Fig. 2 respectively. All sharp corners in the model geometry were filleted with the radius of 5 μm to avoid singularity problems. The mercury contact angle^3-4^ was set to 140°. During the bubble collapse process the radius of the initial curvature of the mercury-air interface was preset to 65.27 μm in order to fit the mercury contact angle of 140° in a 100 μm thick channel. The initial pressure was modified to take into account the Laplace pressure. The Laplace pressure is determined from the Young-Laplace equation as shown in equation (1).

| $\Delta P=P_{inside}-P_{outside}=\gamma(\frac{1}{R_{1}}+\frac{1}{R_{2}})$ | (1) |
| --- | --- |

where *P_inside_* is the pressure inside the conductive liquid, P_outside_ is the pressure outside the conductive liquid (i.e. the pressure of dielectric fluid), and γ is the surface tension of the conductive liquid. R_1_ and R_2_ are the radii of curvature for each of the axes parallel to the surface. In our study the bubble growth and collapse simulation was performed in two ways, i.e. as a simulation of both growth and collapse treated as one process and as a simulation performed in two parts, with growth and collapse treated as two separate processes. We have not observed any substantial differences between these two approaches. The summary of the model parameters are given in Table 1


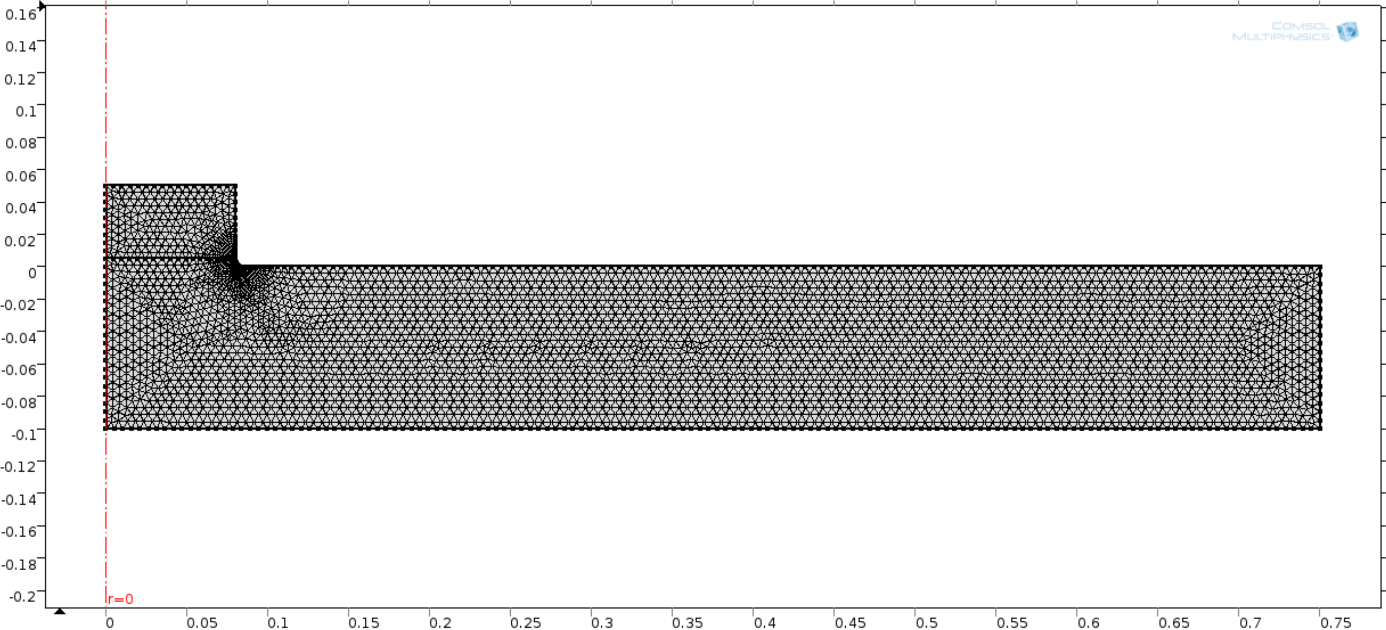


FIG. 1: Mesh used in bubble growth simulation


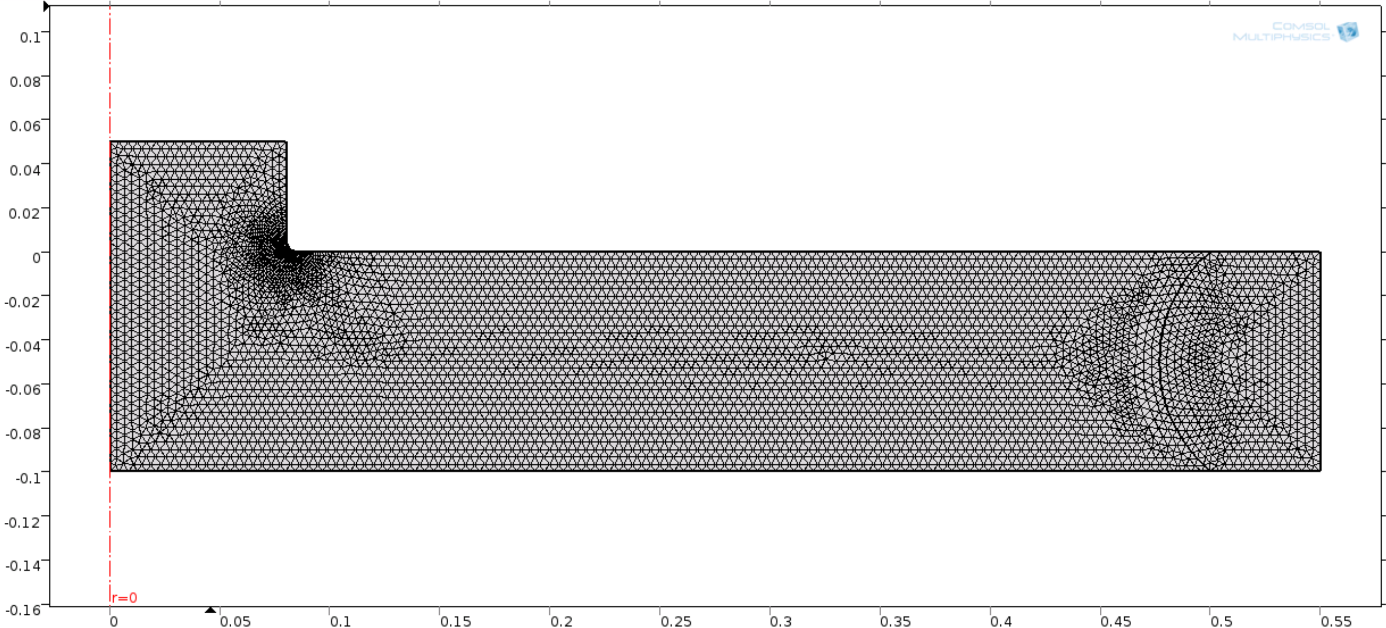


FIG. 2: Mesh used in the bubble collapse simulation

| Parameters | Bubble growth | Bubble collapse |
| --- | --- | --- |
| Flow model | 2D axisymmetric 2 phase laminar flow | 2D axisymmetric 2 phase laminar flow |
| COMSOL study mode | Transient | Transient |
| Air inlet radius | 80 μm | 80 μm |
| Total working area radius | 750 μm | 550 μm |
| Gap | 100 μm | 100 μm |
| Contact angle | 140° | 140° |
| Surface tension | Build-in parameter (Hg-Hg vapor) | Build-in parameter (Hg-Hg vapor) |
| Maximum element size | 5.25e-3 mm | 4.2e-3 mm |
| Minimum element size | 1.5e-4 mm | 6e-5 mm |
| Maximum element growth rate | 1.13 | 1.1 |
| Curvature factor | 0.3 | 0.25 |
| Resolution of narrow regions | 1 | 1 |

Table 1: Detailed parameters of COMSOL model

**Energy harvesting from machine motion**

An application example, dealing with machine motion is shown in Fig. 3. The energy harvesting device is located at the point where the rod string is attached to an oil pump head. It can be utilized to power the pump load cell and associated equipment. As the pump head moves up and down (about 0.2 Hz frequency) the force acting on the harvester top plate from the rod string alternates, causing periodic compression of the bellow chamber and displacing the dielectric fluid contained in the chamber through the REWOD chip, producing electrical power. The displaced fluid is accumulated in the bottom housing chamber and is periodically returned back to the top chamber through an auxiliary bypass check valve completing the cycle. Estimations, as discussed below, show that such a device can generate in excess of 5 W of usable electrical power.


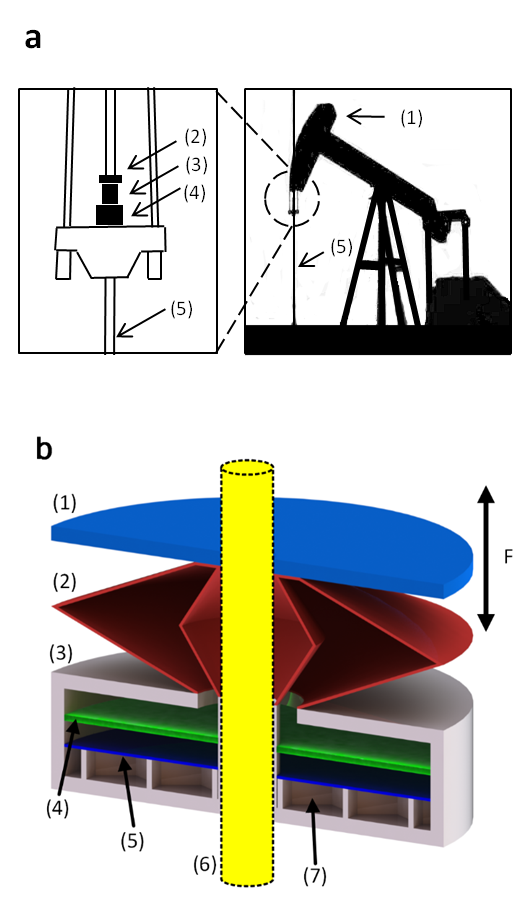


**Figure 3 |** **Energy harvesting from machine motion.** (a) Schematics of oil pump and the possible location where to place the bubbler; (1) indicates pump head, (2) rod clamp, (3) energy harvester, (4) load cell, and (5) rod string. (b) The schematics of the bubbler showing (1) top plate, (2) bellow chamber, (3) housing chamber, (4) bubbler, (5) elastic membrane, (6) rod string, and (7) gas chamber.

**References**

1. <http://www.comsol.com/>
2. Abell, A., Willis, K. & Lange, D. Mercury intrusion porosimetry and image analysis of cement-based materials. *Journal of Colloid and Interface Science* **211**, 39-44, doi:10.1006/jcis.1998.5986 (1999).
3. Ellison, A., Klemm, R., Schwartz, A., Grubb, L. & Petrash, D. Contact angles of mercury on various surfaces and effect of temperature. *Journal of Chemical and Engineering Data* **12**, 607-609, doi:10.1021/je60035a037 (1967).
4. Smithwick, R. Contact-angle studies of microscopic mercury droplets on glass. *Journal of Colloid and Interface Science* **123**, 482-485, doi:10.1016/0021-9797(88)90269-X (1988).

**Video Legend**

**Supplementary video 1 |** The animation of the bubbler operation.

**Supplementary video 2 |** The animation of the fluidic dynamics simulation results representing one full cycle of bubble growth and collapse.

**Supplementary video 3 |** The high-speed camera video showing the processes of bubble growth and collapse
